# Genome Sequence of *Prosthecochloris* sp. Strain HL-130-GSB, from the Phylum *Chlorobi*

**DOI:** 10.1128/genomeA.00538-17

**Published:** 2017-06-15

**Authors:** Vera Thiel, Daniela I. Drautz-Moses, Rikky W. Purbojati, Stephan C. Schuster, Stephen Lindemann, Donald A. Bryant

**Affiliations:** a Department of Biochemistry and Molecular Biology, The Pennsylvania State University, University Park, Pennsylvania, USA; b Department of Biological Sciences, Tokyo Metropolitan University, Hachioji, Tokyo, Japan; c Singapore Centre for Environmental Life Sciences Engineering, Nanyang Technological University, Singapore, Singapore; d Biological Sciences Division, Pacific Northwest National Laboratory, Richland, Washington, USA; e Department of Food Science, Whistler Center for Carbohydrate Research, Purdue University, West Lafayette, Indiana, USA; f Department of Chemistry and Biochemistry, Montana State University, Bozeman, Montana, USA

## Abstract

The genome of the green sulfur bacterium *Prosthecochloris* sp. strain HL-130-GSB, isolated from a cyanobacterial mat obtained from Hot Lake, a saline meromictic lake in Washington, USA, comprises 2,437,774 bp in a single contig. The genome is predicted to encode 2,565 proteins and contain 47 tRNA genes and 2 rRNA operons.

## GENOME ANNOUNCEMENT

*Prosthecochloris* sp. strain HL-130-GSB is a slightly thermotolerant, anaerobic, photoautotrophic green sulfur bacterium (GSB; phylum *Chlorobi*, family *Chlorobiaceae*). Strain HL-130-GSB was isolated from a microbial mat obtained from Hot Lake (Washington, USA; 48°58′23″N, 119°28′35″W), a shallow, meromictic salt lake dominated by MgSO_4_ (1, 2). The mat sample HL6812-130 was obtained from a depth of 55 cm, beneath the chemocline at a water temperature of 24°C on 8 June 2012. The strain was isolated using Pfennig’s medium supplemented with “Hot Lake salts,” containing 0.4 M MgSO_4_, 0.08 M Na_2_SO_4_, and 20 mM KCl, as well as 0.05% (wt/vol) Mg/NH_4_-acetate mix (1:1) in agar shakes in the light at room temperature. Growth was observed up to 45°C. Two rRNA operons were present in the genome; the 16S rRNA genes were identical and shared 99.5% nucleotide identity to those of *Prosthecochloris* sp. strain CHP 3401, which was isolated from a hypersaline lake in Spain (3). This strain is considered to represent an undescribed species with sequence similarities of 96.9% to three closest type strains, *Prosthecochloris aestuarii* DSM 271 (4), *Prosthecochloris vibrioformis* DSM 260 (5, 6), and *Prosthecochloris indica* JAGS6 (7).

Purified genomic DNA from *Prosthecochloris* sp. strain HL-130-GSB was sequenced using a PacBio RSII instrument in one single-molecule real-time (SMRT) cell that yielded 927,499,821 bp (91,952 subreads) and was assembled with the HGAP3 (8) workflow in the SMRT Analysis 2.3.0 package (https://github.com/PacificBiosciences/smrtmake). The assembly used for genome analysis contained a single contig with a length of 2,437,774 bp, with an average G+C content of 52% and mean coverage of 260-fold. Both the G+C content and genome size are similar to those of other phototrophic *Chlorobi* (9).

Annotation using RAST (10) predicted 2,565 protein-coding genes, 47 tRNA genes, and 2 rRNA operons. AmphoraNet (11) identified all 31 phylogenetic marker genes, which confirmed the phylogenetic assignment of this organism to the *Chlorobiaceae*.

Consistent with the photoautotrophic growth characteristics of this and other GSB, the genome encodes a type-1 photosynthetic reaction center (*pscABCD*), the bacteriochlorophyll *a*-binding Fenna-Matthews-Olson protein (*fmoA*), and chlorosomes (*csmABCDFHIJX*), as well as the diagnostic enzymes for the reductive tricarboxylic acid (TCA) cycle (*aclAB, kor,* and *por*). The strain contains photosynthetic pigments bacteriochlorophyll (BChl) *a*, *c*, and *d* (12) and carotenoids of the chlorobactene series. All genes necessary for the biosynthesis of these pigments (*bchDEHIJM, bciABC, bchBLN, bchXYZ, bchCFG, chlG, bchKQRUV, crtBCPHQU,* and *cruACD*) are present. The presence of genes encoding nitrogenase (*nifABDEHKNV*) indicates the ability to fix dinitrogen (13, 14). The genome contains genes for dissimilatory sulfur oxidation (*sqr, dsrABCEFHJKLMNOP*, and 2 genes encoding PSRLC3 [13]) but lacks genes for assimilatory sulfate reduction. Thiosulfate utilization was not observed, and a *sox*-type gene cluster for thiosulfate oxidation was absent (13, 15). Similar to other, mostly marine, GSB, strain HL-130-GSB contains genes encoding an Na^+^-transporting electron transport complex (*rnfABCDEG*), an Na^+^-transporting NADH:ubiquinone oxidoreductase (*nqrABCDEF*), and a multisubunit Na^+^/H^+^ antiporter (*mnhABCDEFG*) (13). The presence of numerous genes for oxidative stress response (e.g., *cydAB, nox, roo, bcp-1, bcp-2, tpx-2, sodB,* and *msrA*) is consistent with observations for other members of this group of strictly anaerobic bacteria (13).

### Accession number(s).

The genome has been deposited at DDBJ/EMBL/GenBank under the accession no. CP020873.
